# Eicosapentaenoic Acid (EPA) and Docosahexaenoic Acid (DHA) Ameliorate Heart Failure through Reductions in Oxidative Stress: A Systematic Review and Meta-Analysis

**DOI:** 10.3390/antiox13080955

**Published:** 2024-08-06

**Authors:** Jayant Seth, Sohat Sharma, Cameron J. Leong, Simon W. Rabkin

**Affiliations:** 1 Faculty of Medicine, University of British Columbia, 9th Floor 2775 Laurel St., Vancouver, BC V5Z 1M9, Canada; jayseth@student.ubc.ca (J.S.); sohat410@student.ubc.ca (S.S.); camleong@student.ubc.ca (C.J.L.); 2 Department of Medicine, Division of Cardiology, University of British Columbia, 9th Floor 2775 Laurel St., Vancouver, BC V5Z 1M9, Canada

**Keywords:** eicosapentaenoic acid, docosahexaenoic acid, oxidative stress, heart failure

## Abstract

The objectives of this study were to explore the role that eicosapentaenoic acid (EPA) and/or docosahexaenoic acid (DHA) plays in heart failure (HF), highlighting the potential connection to oxidative stress pathways. Following PRISMA guidelines, we conducted electronic searches of the literature in MEDLINE and EMBASE focusing on serum EPA and/or DHA and EPA and/or DHA supplementation in adult patients with heart failure or who had heart failure as an outcome of this study. We screened 254 studies, encompassing RCTs, observational studies, and cohort studies that examined HF outcomes in relation to either serum concentrations or dietary supplementation of EPA and/or DHA. The exclusion criteria were pediatric patients, non-HF studies, abstracts, editorials, case reports, and reviews. Eleven studies met our criteria. In meta-analyses, high serum concentrations of DHA were associated with a lower rate of heart failure with a hazard ratio of 0.74 (CI = 0.59–0.94). High serum concentrations of EPA also were associated with an overall reduction in major adverse cardiovascular events with a hazard ratio of 0.60 (CI = 0.46–0.77). EPA and DHA, or n3-PUFA administration, were associated with an increased LVEF with a mean difference of 1.55 (CI = 0.07–3.03)%. A potential explanation for these findings is the ability of EPA and DHA to inhibit pathways by which oxidative stress damages the heart or impairs cardiac systolic or diastolic function producing heart failure. Specifically, EPA may lower oxidative stress within the heart by reducing the concentration of reactive oxygen species (ROS) within cardiac tissue by (i) upregulating nuclear factor erythroid 2-related factor 2 (Nrf2), which increases the expression of antioxidant enzyme activity, including heme oxygenase-1, thioredoxin reductase 1, ferritin light chain, ferritin heavy chain, and manganese superoxide dismutase (SOD), (ii) increasing the expression of copper–zinc superoxide dismutase (MnSOD) and glutathione peroxidase, (iii) targeting Free Fatty Acid Receptor 4 (Ffar4), (iv) upregulating expression of heme-oxygenase-1, (v) lowering arachidonic acid levels, and (vi) inhibiting the RhoA/ROCK signaling pathway. DHA may lower oxidative stress within the heart by (i) reducing levels of mitochondrial-fission-related protein DRP-1(ser-63), (ii) promoting the incorporation of cardiolipin within the mitochondrial membrane, (iii) reducing myocardial fibrosis, which leads to diastolic heart failure, (iv) reducing the expression of genes such as Appa, Myh7, and Agtr1α, and (v) reducing inflammatory cytokines such as IL-6, TNF-α. In conclusion, EPA and/or DHA have the potential to improve heart failure, perhaps mediated by their ability to modulate oxidative stress.

## 1. Introduction

The role of long-chain ω-3 fatty polyunsaturated acids in heart failure is controversial. Docosahexaenoic acid (DHA) and eicosapentaenoic acid (EPA) are both omega-three fatty acids that are found in high concentrations in certain fish and are widely used as supplements. Low plasma levels of DHA or EPA appear to be associated with adverse long-term prognosis in patients with heart failure, while high plasma levels are associated with a better prognosis [1,2]. Randomized control trials administering EPA and or DHA to patients with established coronary artery disease or at high risk for cardiovascular disease have usually reported no benefit in these patient populations [3,4,5], although there are exceptions [6]. However, most of these studies focused on cardiovascular outcomes and did not focus on heart failure (HF) as an entry criterion or a primary outcome. Heart failure is not a single clinical entity with a single etiology. Heart failure has been categorized according to an individual’s ejection fraction, into those with reduced ejection fraction (less than 40% or HFrEF), heart failure with preserved or normal ejection fraction (HFpEF), and heart failure with midrange ejection fraction. HFrEF can be due to a variety of causes, including ischemia (coronary artery disease) with or without myocardial infarction and cardiomyopathy to mention a few. HFpEF is associated with a wide range of conditions and has been further subtyped into as many as six different kinds [7]. Whether trials of ω-3 fatty acid supplementation have considered the types of heart failure is unclear.

Several lines of evidence are required to establish a relationship between a risk factor and a disease [8]. These include demonstrating an association between EPA and/or DHA and heart failure, determining the existence of a gradient of risk between low levels of EPA and or DHA and heart failure, and establishing a reduction in heart failure with higher EPA/DHA administration. Further, there should be biological linkage or evidence to suggest a credible biological mechanism. In clinical studies as well as animal models of HF, increased levels of ROS and impairment of antioxidant defenses have been identified and correlated with cardiac systolic and diastolic dysfunction [9,10,11].

The objective of this study was to examine studies that reported serum levels of EPA and/or DHA rather than infer EPA/DHA circulating levels from dietary intake of fish. It further focused only on studies that examined heart failure. This study found evidence to link EPA and/or DHA with heart failure and proposes a novel connection between these fatty acids and heart failure that involves oxidative stress.

## 2. Materials and Methods

This review was directed following the 2020 Preferred Reporting Items for Systematic Reviews and Meta-Analyses (PRISMA) guidelines [12]. Electronic searches were conducted in MEDLINE and EMBASE with the following search strategy: ((“Eicosapentaenoic Acid” [Mesh]) OR “Docosahexaenoic Acids” [Mesh]) AND (“Heart Failure” [Mesh] OR “Heart Failure, Diastolic” [Mesh] OR “Heart Failure, Systolic” [Mesh])’ from database inception to April 2024.

The inclusion criteria were cohort studies or all primary randomized controlled trials published in English that examined the effect of serum EPA and/or DHA or EPA and/or DHA supplementation in adult patients with heart failure or who had heart failure as an outcome of this study. The exclusion criteria were papers that involved pediatric patients or non-HF studies. Abstracts, editorials, case reports, and reviews were also excluded. As there were no primary data collected, there was no requirement for our ethics committee to review this study.

All references were uploaded to Covidence and were electronically merged to remove duplicates [13]. Two authors individually reviewed each study to determine their inclusion or exclusion. Upon searching references of publications, additional articles regarding left ventricular assessment after EPA/DHA administration in HF patients were added to the review. The data extracted from each study were study design, country in which the study was conducted, sample size, mean age, % male, duration of follow-up, study design, polyunsaturated fatty acid (PUFA) treatment (EPA, DHA, others), mass of fatty acid administered, duration of fatty acid regime, type of heart failure as per left ventricular ejection fraction, survival data, and hazard ratios. Two reviewers (JS and SS) conducted data extraction, and a consensus was reached for any conflicts, as any conflicts were resolved by agreement between JS and SS.

## 3. Results

**Figure 1:**
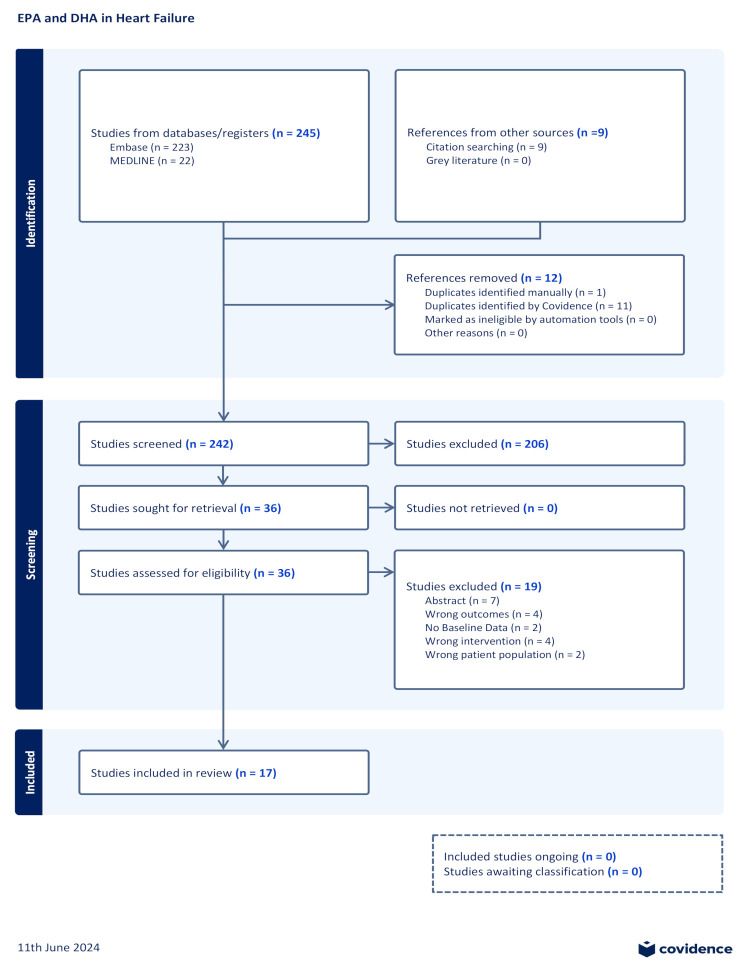
PRISMA flow diagram.

Two hundred and forty-five studies were uploaded onto Covidence for screening from the literature search. Two hundred and three studies were identified after duplicates were removed. Nine additional studies were added through citation search. Two hundred and six abstracts were excluded, as they did not meet the inclusion criteria; mainly, they were not the correct population, most notably pediatric patients, or they did not have heart failure, did not use EPA or DHA, or did not indicate a heart-failure-related outcome. After abstract exclusion, 36 studies were examined for full-text review. Of these, 17 studies were included for data extraction. Seven studies were excluded as they were conference proceedings with no manuscript. Four studies were excluded as they did not examine heart failure outcomes. Two studies were excluded as they did not include baseline values for heart failure outcomes. Four studies were excluded and deemed to have a wrong intervention, as they did not measure or supplement with EPA and/or DHA. Finally, two studies were excluded because they examined pediatric populations. The study selection process is illustrated in Figure 1.

The characteristics of the 17 studies that examined EPA/DHA in heart failure show a diverse range (Table 1).

Jiang et al. evaluated the prognostic value of ω-3 fatty acids in patients with heart failure [14]. The study measured serum levels of ω-3 fatty acids as a percent of total fatty acid weight. In a sample of 109 patients, median EPA and DHA percent compositions were 0.45% and 3.07%, respectively. Low and high values were, respectively, the 25th percentile and 75th percentile. High EPA was significantly associated with survival (hazard ratio [HR] = 0.73, 95% CI = 0.573–0.972). DHA was associated with increased survival as well (HR = 0.67, 95% CI = 0.43–1.04). 

Mozafferian et al. enrolled 2735 patients without heart disease and evaluated the relationship between serum ω-3 fatty acids and the incidence of heart failure [15]. They found that serum EPA was inversely correlated with HF development. The risk was approximately 50% lower in the highest versus the lowest quartile (hazard ratio [HR], 0.52 [95% CI, 0.38 to 0.72]; *p* for trend = 0.001. Similarly, DHA showed a low HR of 0.84 [CI, 0.58 to 1.21]; *P* for trend = 0.38. 

Hara et al. evaluated whether or not serum levels of EPA and DHA correlated with heart-failure-free survival and HF hospitalization after myocardial infarction [16]. EPA and DHA serum levels were divided into tertiles. Both low EPA and DHA groups had worse heart-failure-free survival and more heart failure hospitalization. DHA- and EPA-Low groups showed significantly worse HF-free survival (hazard ratio (HR) 1.68, 95% confidence interval (CI) 1.03–2.72, *p* = 0.0358, and HR 1.69, 95% CI 1.05–2.72, *p* = 0.0280, respectively), with the EPA-Low group having a higher risk of HF hospitalization (HR 2.40, 95% CI 1.21–4.75, *p* = 0.0097) than the DHA-Low group (HR 1.72, 95% CI 0.86–3.45, *p* = 0.1224).

Ouchi et al. examined the association of serum DHA and long-term mortality in patients with acute decompensated heart failure [2]. Serum concentrations were measured once. Median EPA was 40.2 ug/mL, and DHA was 109.5 ug/mL. Patients were subsequently analyzed as those above and below the median. The event-free survival rates for all-cause death were significantly (*p* < 0.05) better (higher) in patients with high PUFA levels than in those with low PUFA levels. 

Block et al. carried out a clinical trial to assess whether or not plasma concentrations of EPA would predict the incidence of heart failure [17]. Cox proportional hazard modeling was used to estimate hazard ratios associated with plasma phospholipid %EPA on a log scale. Median %EPA was 0.70% for all participants. Patients were subsequently analyzed as those above and below this median. They found that log% EPA was associated with lower HF incidence (hazard ratio: 0.73 [95% CI 0.60 to 0.91] per log-unit difference in %EPA; *p* = 0.001).

In a retrospective study, Matsuo et al. found that serum concentrations of DHA were associated with all-cause mortality in patients with heart failure with preserved ejection fraction [1]. Serum concentrations were measured once. Median EPA and DHA were identified as, respectively, 46.3 ug/mL and 115.8 ug/mL. Patients were subsequently analyzed as those above and below the median. Multivariate regression analysis revealed that DHA levels were significantly associated with a lower incidence of all-cause death (HR: 0.16, 95% CI: 0.06–0.44, *p* = 0.001).

In an observational study, Le et al. compared EPA and DHA serum levels in patients [18]. They found that compared with the first quartile, in the fourth quartile, EPA was associated with lower MACE (HR = 0.36 (CI: 0.22, 0.58), including HF hospitalization. However, DHA was not associated with a lower risk of MACE. 

Selvaraj et al. analyzed the REDUC-IT trial and found that icosapent ethyl reduced MACE in the subset of individuals with heart failure (HR = 0.75, CI = 0.68–0.83) [19].

Kohashi et al. showed that EPA treatment of patients with heart failure significantly reduced MACE (HR = 0.21, CI = 0.05 = 0.93) [20]. It was a nonrandomized observational study where 139 patients with congestive heart failure were allocated into either an EPA or non-EPA group. Patients with dyslipidemia at baseline were allocated to the EPA group.

Several studies examined left ventricular systolic function in persons receiving EPA, DHA, or combinations of n3-PUFA. In the study by Kohashi et al., the group given EPA after 12 months showed an improvement in LVEF [20].

Ghio et al. randomized 312 patients to n3-PUFA administration, but the composition of EPA and DHA was not clearly stated. They found that at one year of treatment, LVEF improved from 30.0% (CI = 29.0–31.0) to 32.5% (95% CI = 31.4–33.5). After 3 years, LVEF improved further to 33.5 (CI = 32.0–34.9) [21]. 

Nodari et al. carried out a double-blind randomized control trial in which they administered both 1 g/day or 4 g/day of n3-PUFA and found that both doses increased LVEF after 3 months [22]. The changes were statistically significant, with LVEF improving from 24 ± 8% to 27 ± 8% (SD) in the 1 g/day arm (*p* = 0.01) and from 24 ± 7% to 29 ± 8% (SD) (*p* = 0.005).

Kojuri et al. carried out a double-blind randomized control trial in 70 patients with congestive heart failure [23]. A total of 38 patients received n3-PUFA, while 32 patients received a placebo. This double-blind randomized control trial was conducted in Iran over a one-year period. They found that administration of n3-PUFA reduced left ventricular mass and improved ejection fraction but nonsignificantly.

Chrysohouu et al. enrolled 205 consecutive patients with chronic compensated heart failure due to ischemic heart disease or dilated cardiomyopathy, with NYHA classification I-III, under optimal medical treatment [24]. These patients were one-to-one randomized to a 1000 mg omega 3-PUFA supplementation or no supplementation. They found that *n*-3 PUFA supplementation improved left ventricular diastolic function and subsequently improved LVEF.

Nodari et al. enrolled 47 patients and administered tablets of 1.0 g gelatin capsules containing 850 to 882 mg of eicosapentaenoic acid [EPA] and docosahexaenoic acid [DHA] ethyl esters in the average ratio EPA/DHA of 0.9:1.5 [25]. They found that compared with patients on placebo, those receiving *n*-3 PUFAs showed a decrease from baseline in the LV end-systolic volume from 120 ± 53 (SD) to 111 ± 52 mL versus from 122 ± 37 to 122 ± 36 mL on placebo, (*p* = 0.039) with a concomitant increase in the LVEF, from 36 ± 9% to 39 ± 10% versus from 36 ± 10% to 34 ± 9% with placebo (*p* = 0.01) and a slight but significant increase in peak VO_2_ (from 19.6 ± 3.9 to 20.1 ± 2.7 mL/kg/min versus from 16.3 ± 4.2 to 16.4 ± 3.9 mL/kg/min with placebo, *p* = 0.03).

Moertl et al. enrolled 43 patients with severe nonischemic heart failure and treated either with 1 g/d n3-PUFA (*n* = 14), 4 g/d n3-PUFA (*n* = 13), or placebo (*n* = 16) for 3 months [26]. They found that patients who received n3-PUFA experienced significant increases in LVEF. Additionally, they found that these patients experienced parallel improvements in endothelial function and a slight reduction in serum IL-6 levels.

Zhao et al. carried out a randomized control trial in which they randomized 76 patients with heart failure to receive 2 g/day of *n*-3 PUFA or placebo for 3 months [27]. Treatment with *n*-3 PUFA significantly decreased plasma levels of tumor necrosis factor, interleukin-6, intercellular adhesion molecule 1, and NT-proBNP. The left ventricular ejection fraction showed a small, nonsignificant improvement.

Radaelli et al. administered 2 g n3-PUFA or placebo to patients who experienced myocardial infarction and found that n3-PUFA did not cause a significant improvement in LVEF; however, they did indicate that n3-PUFA improved heart rate variability in patients with congestive heart failure [28].

**Figure 2:**
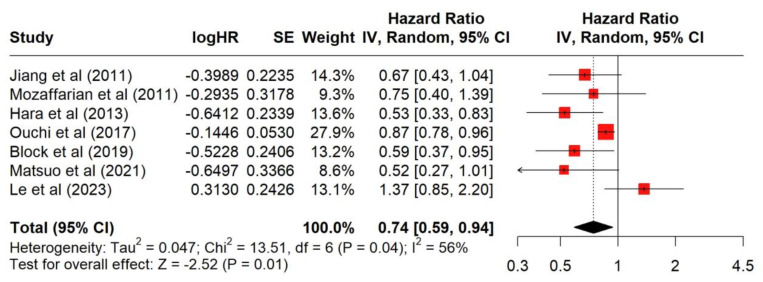
Forest plot of serum DHA concentration and its association with major adverse cardiac events in patients with heart failure [1,2,14,15,16,17,18].

Figure 2 shows that seven studies examined the association of higher DHA serum concentrations with MACE hazard ratios in patients with heart failure. Higher DHA serum concentrations were associated with a significant (*p* = 0.01) reduced hazard of a MACE outcome, with a combined hazard ratio of 0.74 (95% CI: 0.59,0.94). The I^2^ value of 56% suggests that there was moderate heterogeneity among the studies. The *p*-value for heterogeneity (0.04) suggests that the variation between study results is not due to random chance.

**Figure 3:**
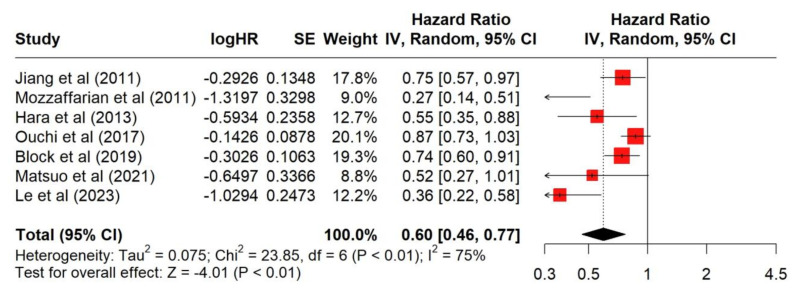
Forest plot of serum EPA concentration and its association with major adverse cardiac events in patients with heart failure [1,2,14,15,16,17,18].

Figure 3 shows that seven studies examined the association of higher EPA serum concentrations with MACE hazard ratios in patients with heart failure. Higher EPA serum concentrations were associated with a significant (<0.01) reduction in the hazard of a MACE outcome with a combined hazard ratio of 0.60 (95% CI: 0.46–0.77). The I^2^ value of 75% suggests that there was significant heterogeneity among the studies. The *p*-value for heterogeneity (<0.01) suggests that the variation between study results is not due to random chance.

**Figure 4:**
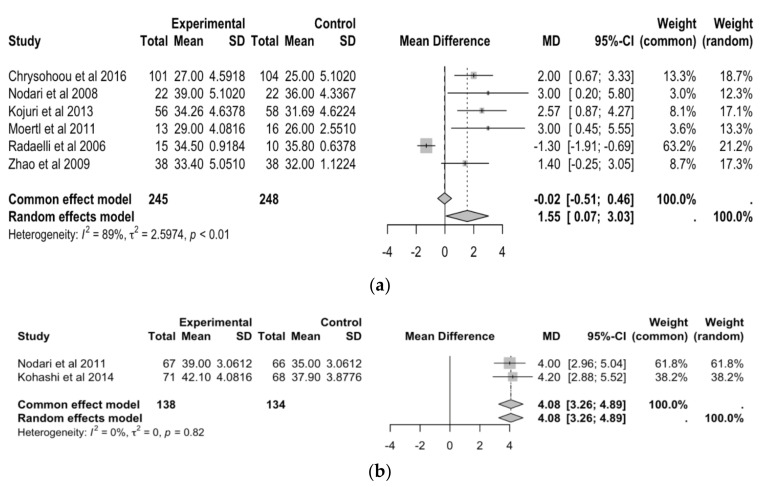
(**a**) Forest plot of mean differences in left ventricular ejection fraction before and after N3-PUFA supplementation. (**b**) Forest plot of mean Differences in left ventricular ejection fraction before and after EPA and/or DHA supplementation [20,22,23,24,25,26,27,28].

Figure 4a shows that six studies examined mean differences in LVEF after n3-PUFA supplementation. Overall, the common effect model shows a mean difference of −0.02 (95% CI: −0.51, 0.46), indicating no significant difference in LVEF before and after n3-PUFA supplementation. The random effects model shows a mean difference of 1.55 (95% CI: 0.04, 3.03), indicating that there was a significant increase in LVEF after n3-PUFA supplementation. The random effects model is considered more appropriate than the common effects model. The I^2^ value of 89% suggests there is significant heterogeneity among the studies. The *p*-value for heterogeneity (<0.01) confirms that the variation between study results is not due to random chance.

Figure 4b shows that two studies examined mean differences in LVEF after EPA and/or DHA supplementation. The random effects model shows a mean difference of 4.08 (95% CI: 3.26, 4.89). The common effects model shows a mean difference of 4.08 (95% CI: 3.26, 4.89), indicating a significant increase in LVEF after n3-PUFA supplementation. The fact that the mean difference and confidence interval are the same for both the common and random effect models suggests there is little variability among the studies. This is further confirmed by the I^2^ value of 0%. The *p*-value for heterogeneity (0.82) confirms that the variation between study results may be due to random chance.

## 4. Discussion

Our systematic review suggests that both EPA and DHA improve heart failure outcomes with a significantly lower hazard rate associated with higher serum levels of EPA and or DHA. Prior reviews have assessed the effects of omega-3 fatty acid supplementation on cardiovascular outcomes, which were a composite of myocardial infarction, coronary heart disease, and cardiovascular mortality in the general population [29]. Previous reviews have not focused only on heart failure, a condition with a different pathophysiology from coronary artery disease although it may be linked to underlying coronary artery disease. We further show that studies of EPA or DHA, not combined with other *n*-3- PUFA products, significantly increase LV ejection fraction. Moreover, studies focusing on EPA or DHA alone showed a greater increase in LVEF compared with studies with *n*-3-PUFA generally. The positive effects of EPA and DHA on left ventricular function may account for the benefits EPA and DHA provide on heart failure outcomes.

Considering the data on the role of oxidative stress in the pathophysiology of heart failure, we explored how EPA and DHA interact with oxidative stress mechanisms to improve HF outcomes.

### 4.1. Heart Failure and Oxidative Stress

The electron transport chain (ETC) of mitochondria generates reactive oxygen species (ROS) as a byproduct of cellular respiration. Cells have many antioxidant defense mechanisms, consisting primarily of enzymatic antioxidants such as superoxide dismutase (SOD), glutathione peroxidase (GSHPx), and catalase [30]. When the balance between oxidation and reduction reactions is disrupted, oxidative stress, the deleterious effects of excessive production of ROS relative to the level of endogenous antioxidants, may occur [31]. These consequences of excessive ROS include disruptions in intracellular signaling pathways, redox signaling, cellular dysfunction, and tissue damage. In heart failure (HF), there is an increased production of ROS, leading to mitochondrial DNA (mtDNA) damage, decreased mitochondrial function, and further ROS formation^12^. In clinical studies, as well as animal models of HF, increased levels of ROS and impairment of antioxidant defenses have been identified and correlated with a decrease in cardiac systolic and diastolic dysfunction [9,10,11]. Clinically heart failure is associated with increased levels of oxidative stress and a deficit of antioxidant reserves (as measured by glutathione peroxidase, plasma lipid peroxides, malondialdehyde, vitamin C, and vitamin E levels) [11].

**Figure 5:**
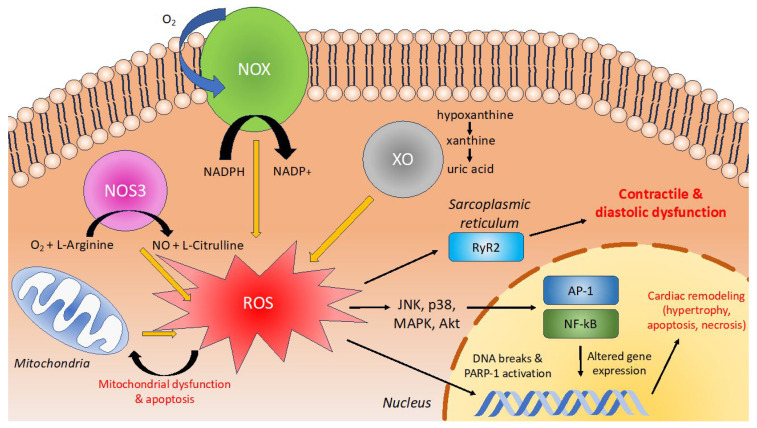
ROS contribute to the pathogenesis of HF in cardiomyocytes. Under normal conditions, nitric oxide synthase (NOS_3_) catalyzes the conversion of O_2_ + L-Arginine ⇌ NO + L-Citrulline. During conditions of oxidative stress, uncoupling of NOS_3_ due to oxidation of BH_4_ leads to the production of harmful ROS. NADPH oxidase is a cytosolic enzyme responsible for the oxidation of NADPH by the equation: NADPH + 2O_2_ ⇌ NADP+ + 2O_2_^−^ + H^+^. This generates two superoxide (O_2_^−^) radicals as a byproduct of the reaction. Xanthine oxidase (XO) first converts hypoxanthine to xanthine, then further oxidizes it to produce uric acid. This is performed by equations: (1) hypoxanthine + H_2_O + O_2_ ⇌ xanthine + H_2_O_2_, and (2) xanthine + H_2_O + O_2_ ⇌ uric acid + H_2_O_2_. Occasionally, mitochondria produce ROS due to the “leakage” of electrons during cellular respiration. This is mainly in the form of O_2_^−^ radicals. Abbreviations: RyR2 = ryanodine receptor 2, ERK = Extracellular signal-regulated kinase. JNK = Jun nuclear kinase, MAPK = mitogen-activated protein kinase, and AP-1 = activator protein-1.

There are several causes for myocardial damage due to oxidative stress which accounts for systolic and diastolic dysfunction and cardiac remodeling (Figure 5). Contractile dysfunction is due mostly to oxidation reactions of thiol groups of the ryanodine receptor (RyR) Ca^2+^ channel leading to aberrant channel opening [32]. This was shown to be reversible when cells were treated with a reducing agent [33]. Secondly, ROS can also cause direct mitochondrial damage and activation of signaling cascades leading to cardiac cell apoptosis and ventricular decompensation [34,35]. Oxidative stress induces aberrant cardiac remodeling, fibroblast proliferation, and matrix remodeling. Oxidative stress can involve myeloperoxidase (MPO). MPO is operative in the production of HFpEF or HFrEF. MPO levels were significantly higher in HFpEF than in individuals without HF [36]. Furthermore, ROS cause DNA breaks and activates nuclear enzyme poly (ADP-ribose) polymerase-1 (PARP-1) which acts as a DNA damage sensor and signaling protein. Increased levels of PARP-1 have been found in animal models of HF, as well as in cardiac biopsies of human patients with HF [37,38]. Furthermore, treatment with PARP-1 inhibitors has therapeutic effects, improving diastolic function, as measured by left ventricular end-diastolic pressure (LVEDP), in a rat model of chronic HF [39].

An emerging hypothesis for the role of ROS in the pathogenesis of HF is the “dark side” hypothesis of long-term β-adrenergic stimulation. Normal β-adrenergic activation leads to the generation of ROS due to increased consumption of oxygen by cardiomyocytes [40]. The “dark side” hypothesis purports that ROS dampen the heart’s short-term response to β-adrenergic stimulation, but prolonged exposure leads to myocardial dysfunction [41]. Treatment of normal human subjects with vitamin C, which modulates ROS, potentiates the inotropic response to dobutamine, a β-adrenergic agonist [42]. The elimination of cardiac ROS by antioxidant treatment led to an increased β-adrenergic response, suggesting that a physiologic role of ROS is to suppress β-adrenergic stimulation. On the other hand, long-term exposure to ROS is detrimental, leading to pathological cardiac remodeling and cardiomyocyte apoptosis [42].

The primary sources of ROS in HF are NADPH oxidases, xanthine oxidase, mitochondria, and uncoupling of nitric oxide synthase 3 (NOS_3_) [43,44]. In guinea pig models of HF and humans with HF, there is increased activity and expression of NADPH oxidase (NOX), which generates ROS [43,44]. Preliminarily studies suggest a link between oxidative stress produced by NOX, and diastolic dysfunction or HFpEF. In a mouse model of myocardial infarction, genetic knockout of NOX subunit p47*^phox^* was protective against LV remodeling and mortality, suggesting NOX contributes to pathological oxidative stress [45]. In a mouse model of diabetes mellitus, treatment with the NOX enzyme inhibitor apomycin was protective not only against ROS production but also diastolic dysfunction and aberrant cardiac remodeling [46,47]. In peripheral blood monocytes of patients with diastolic dysfunction, levels of NOX1 were elevated [48]. Perhaps most intriguingly, Raad et al. [49] studied 75 women with ischemia-induced diastolic dysfunction, measuring aminothiol cysteine levels as markers of oxidative stress. They found that systemic oxidative stress was associated with diastolic dysfunction. Taken together, these studies suggest an association between oxidative stress and diastolic dysfunction and HFpEF.

Xanthine oxidase (XO) dysfunction also plays a role in the pathogenesis of HF. XO, an enzyme that is involved with uric acid synthesis as well as the catabolism of purines, generates ROS as a byproduct of these reactions [50]. Although commonly cited in the literature as a key player in ROS generation during HF, the evidence of the therapeutic effect of XO inhibition is mixed. Initial small clinical trials suggested that allopurinol, an XO inhibitor, increased myocardial efficiency in patients with idiopathic dilated cardiomyopathy or increased LV ejection fraction in heart failure [51]. A much larger RCT of 405 patients treated with either oxypurinol or placebo failed to reproduce these results and found no clinical benefit of XO inhibition [52]. Although XO-mediated ROS production contributes to HF, viable therapeutic strategies are still under active investigation. Adenosine deaminase (ADA), an enzyme that breaks down adenosine and is upstream to XO in the purine degradation pathway, is also observed to have lower activity in heart failure patients [53]. Lowered ADA activity alongside hypoxia in the lowered cardiac output state seen in HF contributes to increased adenosine derivatives in cardiomyocytes [54]. Adenosine derivatives have anti-free-radical effects, suggesting this pathway may serve as a defense mechanism against oxidative stress in heart failure [54].

An important source of ROS in HF is the mitochondrial generation of ROS. Ide et al. [55] evaluated mtDNA damage in a mouse model of HF after MI (left anterior descending coronary artery ligation for 4 weeks). There was an association between ROS and mitochondrial damage (i.e., increased lipid peroxidation, decreased mtDNA copy number, and decrease in mitochondria enzymatic functioning) [55]. These results suggest that HF leads to ROS generation and mitochondrial damage. This sparks a catastrophic cycle of mitochondrial functional decline and further ROS generation.

Finally, the uncoupling of nitric oxide synthase 3 (NOS_3_), which normally produces nitric oxide (NO), has also been implicated in HF [56]. Under conditions of oxidative stress, oxidation of the BH_4_ cofactor may occur. This leads to structural instability of NOS3 and the generation of ROS. Takimoto et al. showed that in a mouse model of HF, a knockout NOS_3_ was protective against diastolic dysfunction [57]. Additionally, treatment of HF mice with BH_4_ prevented uncoupling of NOS_3_ and generation of ROS, reducing the severity of cardiac remodeling. We summarize some of the different pathways by which oxidative stress contributes to heart failure (Figure 5).

### 4.2. Eicosapentaenoic Acid and Oxidative Stress

**Figure 6:**
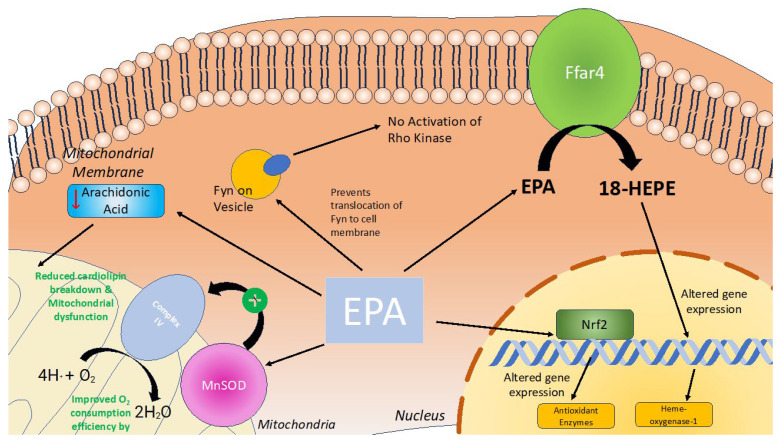
EPA’s role in reducing oxidative stress within cardiomyocytes. Under normal conditions, EPA (eicosapentaenoic acid) is involved in various cellular processes that promote mitochondrial efficiency and protect against dysfunction. During conditions of oxidative stress, EPA is shown to reduce the breakdown of cardiolipin and mitigate mitochondrial dysfunction. It improves O2 consumption efficiency by Complex IV in the mitochondrial membrane, facilitated by MnSOD (manganese superoxide dismutase). EPA also prevents the translocation of Fyn to the cell membrane, thereby inhibiting the activation of Rho Kinase. Additionally, EPA interacts with Ffar4, leading to the production of 18-HEPE. EPA also activates transcription factor Nrf2. This interaction results in altered gene expression through Nrf2 and 18-HEPE, which enhances the expression of antioxidant enzymes, including heme-oxygenase-1.

Eicosapentaenoic acid (EPA) has been proposed to play a role in regulating immune response, inflammation, and oxidative stress, contributing to the beneficial cardiac effects of EPA [58] (Figure 6).

There are several proposed mechanisms by which EPA may lower oxidative stress within the heart. Firstly, EPA exerts antioxidant effects by reducing the concentration of reactive oxygen species (ROS) within cardiac tissue [59]. EPA can both reduce ROS production and increase ROS consumption/breakdown leading to lower steady-state ROS levels. EPA can upregulate nuclear factor erythroid 2-related factor 2 (Nrf2), a transcription factor expressed ubiquitously in the cardiovascular system [59,60]. In turn, Nrf2 increases the expression of antioxidant enzyme activity, including heme oxygenase-1, thioredoxin reductase 1, ferritin light chain, ferritin heavy chain, and manganese superoxide dismutase (SOD) [59]. Within cardiac tissue, EPA and other *n*-3 polyunsaturated fatty acids act at the level of the mitochondria to increase the expression of copper–zinc superoxide dismutase (MnSOD). In vivo experiments in mice demonstrate that increased MnSOD activity exerts cardioprotective effects by improving oxygen consumption efficiency and contractile function [61]. In addition, EPA reduces cardiac myocyte sensitivity to ROS. Zhou et al. demonstrated that in situations of ischemia–reperfusion injury (IRI), EPA increased levels of antioxidant enzymes, such as glutathione peroxidase and SOD, preventing further tissue damage [62].

EPA may also mitigate oxidative stress in the heart by targeting Free Fatty Acid Receptor 4 (Ffar4), a G-protein-coupled receptor for endogenous medium-/long-chain fatty acids that attenuates inflammation [63]. Murphy et al. found that in mice lacking Ffar4 (Ffar4KO), there were transcriptional deficits in cytoplasmic phospholipase A2alpha and oxylipin synthesis, leading to increased oxidative stress, cardiac remodeling, hypertrophy, and contractile dysfunction [64]. Under physiological conditions, Ffar4 is involved in producing EPA-derived proresolving oxylipin 18-hydroxyeicosapentaenoic acid (18-HEPE), which reduces myocardial cell death due to oxidative stress. In addition, Murphy et al. demonstrated that 18-HEPE upregulated the expression of heme-oxygenase-1, which also protected myocardial cells from oxidative stress by increasing the consumption of ROS [64].

EPA’s therapeutic effects to reduce the incidence of heart failure may also be explained by its ability to counteract the left ventricular dysfunction caused by oxidative stress. Raad et al. demonstrated that higher oxidative stress, measured by cysteine levels, was associated with diastolic dysfunction in women even after adjusting for common comorbidities [49]. The role oxidative stress plays in diastolic dysfunction is poorly understood [49]. In a large animal model of heart failure with preserved ejection fraction, basal superoxide production was 3-fold higher than controls [65]. In addition, in states of oxidative stress, myocardial sarcoplasmic proteins are phosphorylated by ROS-sensitive enzymes, leading to increased myofilament Ca^2+^ sensitivity and impaired myocyte relaxation [65]. Finally, oxidative stress decreases nitric oxide levels, which leads to sarcomere stiffening [66,67]. We found that EPA, DHA, and some but not all combinations of *n*-3 PUFA improved left ventricular ejection fraction. The findings are similar to that of Liu et al. [68], except we differentiated studies that specified only EPA or DHA from studies that combined all *n*-3 PUFA studies. Animal studies found that EPA may exert positive ventricular remodeling effects by significantly suppressing phenylephrine- and histone deacetylase p300-induced cardiomyocyte hypertrophy, expression of hypertrophy response genes, and acetylation of histone H3K9 [69]. In addition, EPA allosterically inhibits histones and competitively inhibits acetyl-CoA in myocytes which is the mechanism behind the histone deacetylase p300-induced hypertrophic response [69]. Importantly, EPA (1 g/kg) preserved fractional shortening and prevented MI-induced left ventricular remodeling [69]. Thus, EPA may prevent or delay the worsening of heart failure caused by oxidative stress through positive effects on ventricular remodeling.

EPA may protect against oxidative stress in the heart by preventing mitochondrial dysfunction. Metcalf et al. showed that EPA supplementation lowers arachidonic acid levels in cardiomyocytes [70]. High arachidonic acid levels in diabetic hearts promote cardiolipin breakdown, leading to mitochondrial damage and increased ROS production [71]. Thus, by reducing arachidonic acid, EPA helps prevent mitochondrial damage and oxidative stress in the heart.

EPA may help reduce oxidative stress by inhibiting the RhoA/ROCK signaling pathway, which is crucial for various cellular functions including in the cytoskeleton, which is involved in cell proliferation, migration, and contraction [72]. This pathway is particularly important for cardiomyocyte contractile physiology, enabling myocardial tissue to contract cohesively. The RhoA/ROCK pathway contributes to the pathogenesis of heart failure, due to oxidative stress [73]. In oxidative stress conditions, ROS directly activates and increases RhoA levels [74]. Superoxide radicals enhance ROCK activity [75]. Increased RhoA/ROCK signaling can potentially contribute to heart failure [76]. In addition, increased Rho-associated kinase activity due to oxidative stress may lead to aortic wall stiffness, increasing afterload, and further contributing to heart failure [77]. There is also some evidence that increased RhoA/ROCK activity can lead to cardiomyocyte apoptosis [73]. Nakao et al. found that EPA may inhibit Rho-kinase pathway activation by preventing the cellular translocation of Fyn, an Src protein tyrosine kinase (SrcPTK), and the subsequent activation of SrcPTK by SPC. This leads to the inactivation of ROK and the Rho-kinase pathway [78]. By inhibiting this pathway, EPA may mitigate oxidative stress and its contribution to heart failure. Thus, there are a number of different pathways by which EPA acts within cardiomyocytes to reduce oxidative stress (Figure 6).

Because oxidative stress is operative in heart failure, the role of EPA as an antioxidant may explain, at least in part, its therapeutic potential for heart failure.

### 4.3. DHA and Oxidative Stress

DHA has been suggested to be protective of the myocardium by alleviating mitochondrial dysfunction [79]. LPS-induced myocardial damage leads to a significant increase in ROS [80]. These heightened levels of ROS consume the antioxidant capacity of the mitochondria, leading to oxidative stress, which can produce mitochondrial lipid peroxidation and mtDNA damage [81]. Wang et al. found that DHA improved HL-1 cardiomyocyte viability in LPS-induced cardiotoxicity and alleviated cardiac cell apoptotic cell death perhaps by reducing caspase-3 activity [80]. Moreover, DHA also reduced levels of mitochondrial-fission-related protein DRP-1(ser-63) and mitochondrial fusion protein, demonstrating the ability of DHA to reduce mitochondrial dysfunction and mitochondrial fragmentation [80].

DHA promotes the incorporation of cardiolipin within the mitochondrial membrane [82]. Cardiolipin, a phospholipid component of the inner mitochondrial membrane, plays a crucial role in interacting with numerous components of the mitochondrial respiratory chain and generating the electrochemical gradient required for ATP production [83]. Cardiolipin is a common target of lipid peroxidation from ROS, which are often generated as byproducts of the electron chain [82]. Omega 3 fatty acids such as DHA can induce structural changes in cardiolipin that confer protection against lipid peroxidation [82].

Gui et al. explored the impact of DHA on diabetic cardiomyopathy in rats on a high-fat diet [84]. Reactive oxygen species impair mitochondrial function impacting cellular energy production and mitochondrial dysfunction that can lead to cell death of cardiomyocytes [84]. DHA reverses signs of cardiotoxicity and, in addition, prevents the development of myocardial fibrosis [84]. Additionally, DHA reduced the expression of inflammatory cytokines such as IL-6 and TNF-α, as well as hypertrophy-associated genes such as Appa, Myh7, and Agtr1α [84]. DHA prevented TNF-α-induced VCAM1 expression in endothelial cells and decreased VCAM-1 through the NF-kb pathway in [85]. Both of these cytokines, TNF-α and NF-kB, are also implicated in oxidative stress [86].

**Figure 7:**
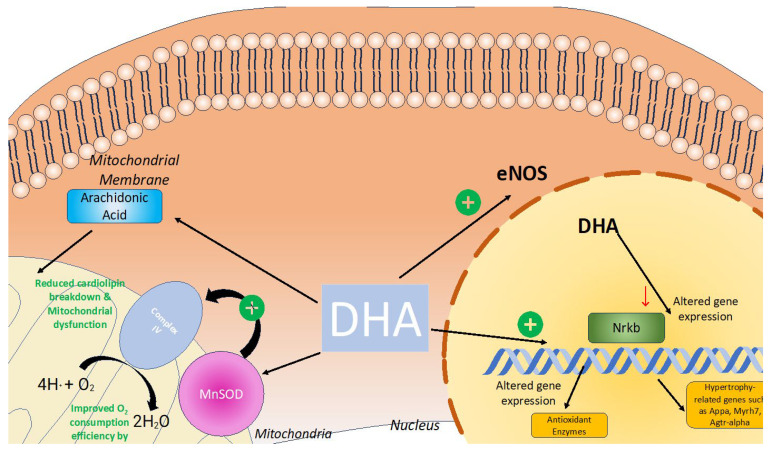
DHA’s role in reducing oxidative stress within cardiomyocytes. DHA plays a role in alleviating oxidative stress within cardiomyocytes. DHA increases the promotion of cardiolipin within the mitochondrial membrane, which subsequently reduces mitochondrial stress and subsequent dysfunction due to oxidative damage. DHA also attenuates the Nrkb pathway and reduces the production of inflammatory cytokines and activation of genes such as appa, Myrh7, and Agtr-alpha, which have been implicated in hypertrophy and fibrosis of cardiomyocytes. DHA also enhances eNOS activity through AKT and HSP90 activation, which subsequently enhances nitric oxide production and minimizes oxidative stress.

In summary, DHA has been shown to have a positive impact on lowering oxidative stress (Figure 7), which subsequently could indicate its therapeutic potential for heart failure.

### 4.4. Strengths, Limitations, and Next Steps

Our review presents several strengths. The studies had large sample sizes with a total of 7469 individuals. Most studies had a long-term follow-up, as all but two studies were tracking patients for a duration of two or more years. This strengthens the quality of evidence for the included observational studies. The analyzed studies were gathered from several countries in different continents. This strengthens the ability to extrapolate the data because of the diverse demographics and pathophysiological etiologies for heart failure.

Several limitations in our study warrant discussion. There are differences in EPA and DHA administration, patient populations with respect to inclusion criteria, and heart failure outcomes. First, observational studies examining serum concentrations of EPA and DHA assess the levels, which can be influenced by dietary intake, metabolism, absorption efficiency, and/or lifestyle considerations. For example, tobacco smokers may have lower circulating *n*-3 long-chain polyunsaturated fatty acids compared with nonsmokers [87]. The prevalence of tobacco usage in different studies may influence the results, but it was not reported consistently in the studies in our meta-analysis. One notable exception was the study by Kohashi et al., which reported comparable percentages of active smokers in both their EPA (14.1%) and non-EPA (16.2%) groups [20]. This minimized the effect of smoking as a confounding variable in their observational study. Another issue is that studies administered varying doses of EPA and DHA, complicating the comparison of heart failure outcomes. Omega-3 fatty acids may not influence cardiovascular outcomes in a simple dose–response manner in all studies. Zhang et al. found a nonlinear dose–response relationship between omega-3 fatty acids and blood pressure, with optimal systolic blood pressure reductions occurring at 2–3 g/day [88].

Another issue is the presence of different comorbidities in the different studies, which could have influenced the response to EPA and DHA. Cardiovascular and metabolic comorbidities such as diabetes, smoking, hypertension, and coronary artery disease increase the risk of developing heart failure. The differing proportions of patients with these comorbidities between studies present potential confounding variables for hazard ratios related to all-cause mortality, major adverse cardiovascular events (MACE), and heart failure incidence. Another issue is whether the beneficial effects of EPA and DHA on heart failure are outweighed by the increased risk of atrial fibrillation in patients who regularly use fish oil supplements, including EPA and DHA [89].

Another limitation is that few studies stratified the effects of EPA and DHA by type of heart failure. While studies reported LVEF, no proportion of patients with HFrEF, HFmrEF, and HFpEF were provided. In addition, there was no consideration on whether EPA and DHA had differential effects on heart failure outcomes when stratifying by systolic, or diastolic heart failure or the underlying etiology of the heart failure. Another consideration is the sex distribution of the study participants. The samples exhibited a disproportionate sex distribution, with a higher representation of males compared with females.

A significant limitation arises from the presence of missing data in follow-up. While all studies conducted thorough follow-ups with respect to HF incidence, cardiovascular events, and all-cause mortality, all the studies did not collect follow-up data on other variables, such as ejection fraction, NYHA class, hospitalization, or NT-pro-BNP. To fully appreciate the potential of EPA and DHA on cardiac function in HF, it is important to corroborate hazard ratios with other numerical factors and assessments of heart function in heart failure.

Furthermore, there were limitations associated with heterogeneity that reduced the reliability of the meta-analyses conducted on both MACE hazard ratios and LVEF mean differences. High heterogeneity among the studies could be due to differences in the study design, populations, interventions, or outcomes measured. This variability compromises the conclusions of the combined effect size in the meta-analyses.

Our study suggests several areas for future research. The next steps to further characterize the role of EPA and DHA in the management of heart failure patients should involve a more thorough evaluation of HF subtypes. There are subtypes of heart failure such as systolic and diastolic heart failure and the different subtypes within each of those kinds of heart failure. We suggest greater female representation in future studies, as this representation will better elucidate sex-based differences in EPA and DHA supplementation, should any differences exist. Furthermore, prespecified randomized clinical trials are still missing, and the use of EPA and DHA is still debated in the treatment of cardiovascular diseases. Currently, European guidelines only suggest the use of icosapent-ethyl, albeit with a low-grade recommendation [90]. Randomized clinical trials must be conducted examining the effect of EPA and DHA supplementation on LVEF, diastolic LV function, cardiac fibrosis, NT-pro-BNP, NYHA classification, and hospitalizations due to HF. This will allow for a more robust quantitative analysis of the impact of EPA and DHA supplementation on heart failure.

### 4.5. Conclusions

In summary, DHA was associated with a lower rate of major adverse cardiovascular events in patients, specifically with heart failure with a hazard ratio of 0.74. EPA also was associated with an overall reduction in major adverse cardiovascular events with a hazard ratio of 0.60. EPA and DHA or n3-PUFA administration was associated with an increased LVEF with a mean difference of 1.55%. There are many cellular mechanisms involving oxidative stress that are likely operative in mediating the beneficial effects of EPA and DHA on heart failure.

## Figures and Tables

**Table 1:** Shows some details of studies examining EPA/DHA in heart failure.

Study	Country	Study Design	%Male	Sample Size	Follow-Up	Age *	Dose EPA/DHA	Dose EPA	Dose DHA
Radaelli et al. 2006	Italy	RCT	96%	15	-	59.4 (2.5)	2 g daily n3-PUFA	-	-
Nodari et al. 2008.	Italy	RCT	95.4%	22	6 months	61.09 ± 11.22	850 to 882 mg of eicosapentaenoic acid [EPA] and docosahexaenoic acid [DHA] ethyl esters in the average ratio EPA/DHA of 0.9:1.5	-	-
Zhao et al. 2009	China	RCT	71%	38	3 months	74 (68,80)	2 g/day of *n*-3 PUFA	-	-
Moertl et al. 2011	Austria	RCT	100%	13	3 months	61.9 ± 9.6	1 g/d omega-3-polyunsaturated fatty acids (n3-PUFAs)	-	-
Jiang et al. 2011	USA	Prospective Cohort Study	57.5%	109	N/A	62 (55, 71)	-	-	-
Nodari et al. 2011	Italy	RCT	95.5%	64	3 months	61 (11.1)	850 to 882 mg of EPA and DHA ethyl esters	-	-
Mozafferian et al. 2012	USA	Prospective Cohort Study	-	2735	14 years	-	-	-	-
Hara et al. 2013	Germany	Retrospective cohort study	-	712	1079 days	65 (57–73)	-	-	-
Kojuri et al. 2013	Iran	RCT	58	38	1 Year	54	2 g/day of omega 3	-	-
Kohashi et al. 2014	Japan	Prospective Cohort Study	-	139	12 months	70.2 ± 9.0	-	1800 mg daily	
Ghio et al. 2014	Italy	RCT	88.8%	312	3 years	67 (11)	1:1 to *n*-3 PUFA (1 g/day)	-	-
Chrysohoou et al. 2016	Greece	RCT	79.2%	101	6 months	63 (12.8)	1000 mg omega 3-PUFA supplementation	-	-
Ouchi et al. 2017	Japan	Prospective Cohort Study	68.3%	306	2.4 ± 1.2 years	66.4 ± 15.0	-	-	-
Block et al. 2019	USA	Prospective Cohort Study	58.9%	292	13 years	69± 9	-	-	-
Matsuo et al. 2021	Japan	Retrospective Cohort Study	42.9%	140	-	84 (77–88)	-	-	-
Selvaraj et al. 2022	USA	RCT	69.3%	1446	-	63.0	-	-	-
Le et al. 2023	USA	Prospective Cohort Study	57%	987	10 years	61.5 ± 12.2	-	-	-

* Values reported as mean ± SD. N/A = Not reported. References [1,2,14,15,16,17,18,19,20,21,22,23,24,25,26,27,28].
